# HtrA1 Is Specifically Up-Regulated in Active Keloid Lesions and Stimulates Keloid Development

**DOI:** 10.3390/ijms19051275

**Published:** 2018-04-24

**Authors:** Satoko Yamawaki, Motoko Naitoh, Hiroshi Kubota, Rino Aya, Yasuhiro Katayama, Toshihiro Ishiko, Taku Tamura, Katsuhiro Yoshikawa, Tatsuki Enoshiri, Mika Ikeda, Shigehiko Suzuki

**Affiliations:** 1 Department of Plastic and Reconstructive Surgery, Japanese Red Cross Fukui Hospital, 2-4-1, Tsukimi, Fukui-City, Fukui 918-8501, Japan; satokoy@kuhp.kyoto-u.ac.jp; 2 Department of Plastic and Reconstructive Surgery, Graduate School of Medicine, Kyoto University, 54 Kawahara-cho, Sakyo-ku, Kyoto 606-8507, Japan; rinok@kuhp.kyoto-u.ac.jp (R.A.); hemim@kuhp.kyoto-u.ac.jp (Y.K.); enotatsu@kuhp.kyoto-u.ac.jp (T.E.); ssuzuk@kuhp.kyoto-u.ac.jp (S.S.); 3 Department of Life Science, Faculty of Engineering Science, Akita University, 1-1 Tegata Gakuenmachi, Akita 010-8502, Japan; hkubota@gipc.akita-u.ac.jp (H.K.); taku@gipc.akita-u.ac.jp (T.T.); 4 Department of Plastic and Reconstructive Surgery, Japanese Red Cross Otsu Hospital, 1-1-35, Nagara, Otsu City, Shiga 520-8511, Japan; ishiko@otsu.jrc.or.jp; 5 Department of Plastic and Reconstructive Surgery, Shiga Medical Center for Adults, 5-4-30, Moriyama, Moriyama City, Shiga 524-8524, Japan; khiro@kuhp.kyoto-u.ac.jp; 6 Department of Plastic and Reconstructive Surgery, Kobe City Medical Center General Hospital, 2-1-1, Minatojima minami-machi, Cyuou-ku, Kobe City, Hyogo 650-0047, Japan; mikaring@crux.ocn.ne.jp

**Keywords:** keloids, fibroproliferative disorder, HtrA1, inflammation

## Abstract

Keloids occur after failure of the wound healing process; inflammation persists, and various treatments are ineffective. Keloid pathogenesis is still unclear. We have previously analysed the gene expression profiles in keloid tissue and found that HtrA1 was markedly up-regulated in the keloid lesions. HtrA1 is a serine protease suggested to play a role in the pathogenesis of various diseases, including age-related macular degeneration and osteoarthritis, by modulating extracellular matrix or cell surface proteins. We analysed HtrA1 localization and its role in keloid pathogenesis. Thirty keloid patients and twelve unrelated patients were enrolled for in situ hybridization, immunohistochemical, western blot, and cell proliferation analyses. Fibroblast-like cells expressed more HtrA1 in active keloid lesions than in surrounding lesions. The proportion of HtrA1-positive cells in keloids was significantly higher than that in normal skin, and HtrA1 protein was up-regulated relative to normal skin. Silencing *HtrA1* gene expression significantly suppressed cell proliferation. HtrA1 was highly expressed in keloid tissues, and the suppression of the *HtrA1* gene inhibited the proliferation of keloid-derived fibroblasts. HtrA1 may promote keloid development by accelerating cell proliferation and remodelling keloid-specific extracellular matrix or cell surface molecules. HtrA1 is suggested to have an important role in keloid pathogenesis.

## 1. Introduction

Keloids are a dermal fibrotic disease characterized by abnormal accumulation of extracellular matrix (ECM) and fibroproliferation in the dermis [1,2]. They appear as raised, red, and inflexible scar tissue that develops during the wound-healing process, even from tiny wounds including vaccination and insect bites. Keloid lesions expand over the boundaries of the initial injury site, and the lesions continue to develop and become larger [3,4]. The many treatments for keloids include steroid injections, steroid tape, and surgery with postoperative irradiation. The cure rate following surgery and postoperative radiation varies widely from 28~89% [3,5,6,7,8] and depends on the individual. Clarifying keloid pathogenesis could improve the treatment outcome.

Previously, we studied the molecular mechanism of keloid pathogenesis using cDNA microarray and Northern blot analysis to compare gene expression patterns in keloid lesions and normal skin [9]. HtrA1, a member of the HtrA family of serine protease and a mammalian homolog of *Escherichia coli* HtrA (DegP), was markedly upregulated in the keloid lesions. As human HtrA1 has multiple domains, including protease, IGFBP, and PDZ domains, HtrA1 has been expected to be a multifunctional protein. Several cellular and molecular studies suggested that HtrA1 plays a key role in regulating various cellular processes via the cleavage and/or binding of pivotal factors that participate in cell proliferation, migration, and cell fate [10,11,12,13] HtrA1 has been suggested to be closely associated with the pathology of various diseases, including osteoarthritis, age-related macular degeneration (AMD), familial cerebral small vessel disease (CARASIL), and malignant tumours. HtrA1 was also suggested to stimulate progression of arthritis through degrading cartilage matrix in osteoarthritis [14]. Recently, the increased expression of human HtrA1 in the mouse retinal pigment epithelium (RPE) was shown to induce vasculogenesis and degeneration of the elastic lamina and tunica media of the vessels, similar to that observed in AMD patients [15,16]. These observations imply that HtrA1 plays a role in the pathogenesis of various diseases by modulating proteins in the ECM or cell surface. Although controversial, HtrA1 has been proposed as a key molecule in osteogenesis and chondrogenesis [14,17,18]. HtrA1 expression is induced during hypertrophic change in chondrocytes, with the up-regulation of the type X collagen marker in keloid lesions [9,18]. HtrA1 is closely concerned with normal osteogenesis and in pathogenesis of arthritis [14]. In arthritis, synovial fibroblasts identified as a major source of HtrA1 degrading cartilage matrix, such as fibronectin and aggrecan, which are abundant in keloid lesions [9,14,18].

Based on the foregoing data, in this study, we focused on HtrA1. We examined the expression and localization of HtrA1 in keloid tissues, using in situ hybridization and immunohistochemical studies. HtrA1 was strongly up-regulated at both the mRNA and protein levels in the hypercellular and active keloid lesions. Silencing *HtrA1* gene expression in keloid fibroblasts significantly inhibited cell proliferation, and additional recombinant HtrA1 stimulated keloid fibroblast proliferation. We propose that HtrA1 may be a pivotal molecule in keloid pathogenesis, and our discussion centres on the possible roles of HtrA1 in the molecular mechanism of keloid development.

## 2. Results

### 2.1. In Situ Hybridization of HtrA1 mRNA in Keloid Lesions and Normal Skin

**Figure 1:**
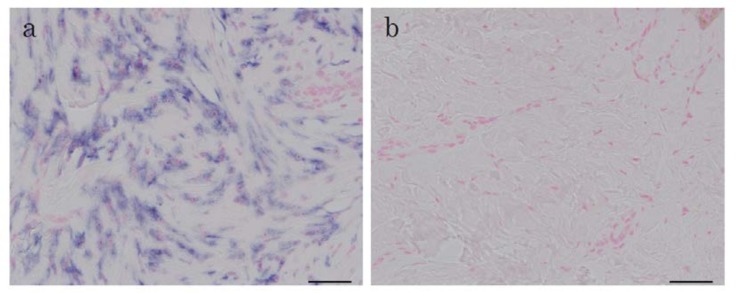
In situ hybridisation for *HtrA1* mRNA in keloid and normal skin. Sections from active keloid lesions (**a**) or unaffected region (**b**) (patient No. 18 in Table 1) were hybridised with a probe specific to *HtrA1* mRNA. Positive signals are visualised in blue. Scale bar = 50 µm.

**Figure 2:**
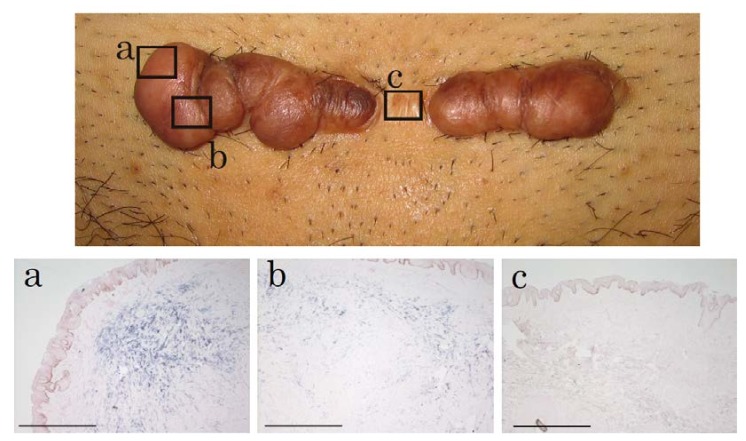
An abdominal keloid after laparoscopic surgery. The activity of keloid was in order a, b and c. Higher activity in regions of the lesion were associated with increased cell proliferation and greater up-regulation of HtrA1. Scale bar = 500 µm.

To confirm the up-regulation of the mRNA level for HtrA1, we previously observed using microarray and Northern blot analyses, and to determine the localization of *HtrA1* mRNA in keloid lesions, in situ hybridization was performed using skin samples from six keloid patients. In one specimen (No. 27 in Table 1), in situ hybridization was performed on several parts of lesions which differed in keloid activity. The expression of the *HtrA1* gene was clearly detected in the fibroblasts in the hypercellular and actively growing area of keloid lesions (Figure 1a, Supplementary Figure S1a,c,e), but not in unaffected skin (Figure 1b). In the sections hybridized with sense probe, no signal was observed (Supplementary Figure S1b,d,f), demonstrating specific staining by the antisense probe. All keloid sections were hard and elevated in the keloid lesions. In these regions, the antisense probe provided strong signals (Figure 1a, Supplementary Figure S1a,c,e). Clinical findings and the results of in situ hybridization of sample 27, which was an abdominal keloid after laparoscopic surgery for removal of uterine myoma, as depicted in Figure 2. Keloid activity was in the order of a, b and c. Higher activity in the affected portion of the lesion was associated with greater cell proliferation and greater up-regulation of HtrA1 (Figure 2). *HtrA1* mRNA was strongly up-regulated, and expression of HtrA1 was more pronounced in keloid lesions.

### 2.2. Immunohistochemical Staining and Western Blot Analysis of HtrA1

**Figure 3:**
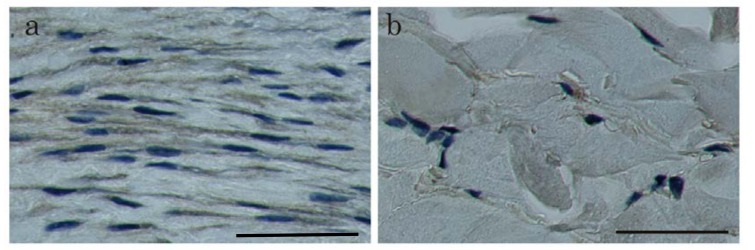
Immunohistochemical staining of HtrA1 protein in keloid (**a**) and normal skin tissue (**b**). Sections from active keloid lesions (**a**) or normal skin (**b**). (**a**) displays the results from patient No. keloid-3 in Table 1, and (**b**) displays the results from patient No. normal skin-1 in Table 1. Positive signals are visualised in brown. Scale bar = 50 µm.

**Figure 4:**
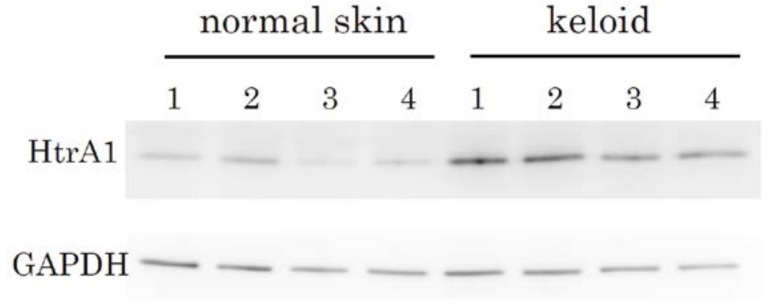
Western blot analysis of HtrA1 in keloid lesions and normal skin tissues. Soluble protein extract (8 µg/lane) was analysed using specific antibodies against HtrA1 or glyceraldehyde-3-phosphate dehydrogenase (GAPDH). Keloid and normal skin samples from four different patients were analysed.

**Figure 5:**
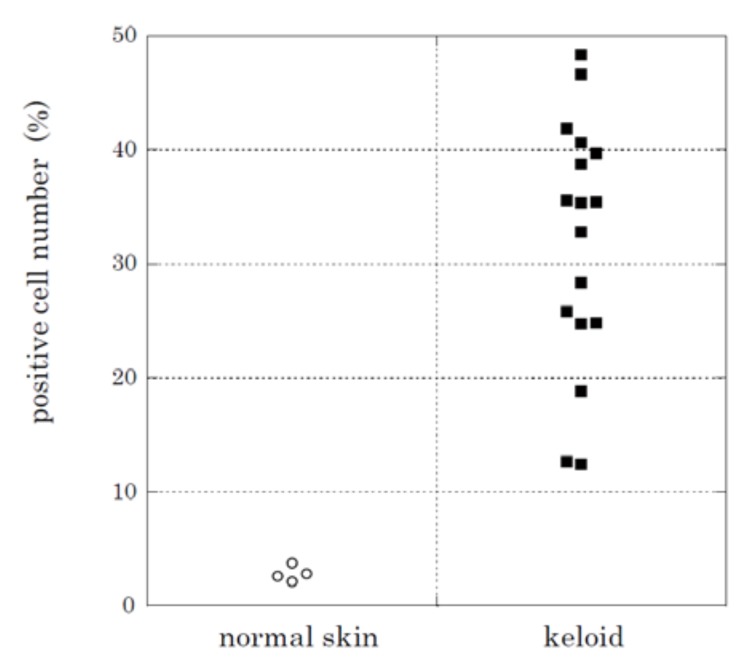
Proportion of fibroblasts expressing HtrA1 protein in keloid lesions and normal skin. The number of fibroblasts with positive signals was counted after immunohistochemical staining of HtrA1 using samples from 17 keloidand 4 unrelated patients. Ten high-power (×400) fields were selected at random from a section and numbers of total and stained fibroblasts were counted. Patient information is described with proportion of HtrA1-positive cells in Table 1.

To examine whether the up-regulation of HtrA1 at the mRNA level leads to increases at the protein level, we performed immunohistochemical analysis to detect HtrA1 (Figure 3a,b, Supplementary Figure S2a–f). HtrA1 was clearly detected by immunostaining in keloids (Figure 3a, Supplementary Figure S2a, c, e, while no signals were observed in normal skin (Figure 3b). These data were consistent with the in situ hybridization findings. No positive signal was found in controls not treated with the primary antibody (Supplementary Figure S2b, d, f). Therefore, HtrA1 was strongly up-regulated at the protein level in active areas of the keloid lesions. To confirm the up-regulation of HtrA1 protein, western blot analysis was performed. In all keloid tissue samples from four patients, HtrA1 protein was up-regulated, relative to four normal skin samples (Figure 4). Enumeration of HtrA1-positive cells after immunohistochemical staining indicated that the proportion of cells expressing detectable levels of HtrA1 in keloid tissue ranged from 12.4% to 48.4%, with an average of 31.9 ± 10.5% (Figure 5). In contrast, the proportion of HtrA1-positive cells in normal skin ranged from 2.1% to 3.8%, with an average of 2.8 ± 0.6%. The proportion of HtrA1-positive cells was significantly higher in keloids than in normal skin (*p* < 0.001). The total number of fibroblasts was much less in normal skin relative to keloid tissue (Figure 3), as previously reported [9]. These results indicate that keloid tissue exhibits an increase in the number of fibroblasts producing HtrA1, as well as an increase in the total number of fibroblasts.

### 2.3. HtrA1 Knockdown Inhibits Keloid Cell Proliferation

**Figure 6:**
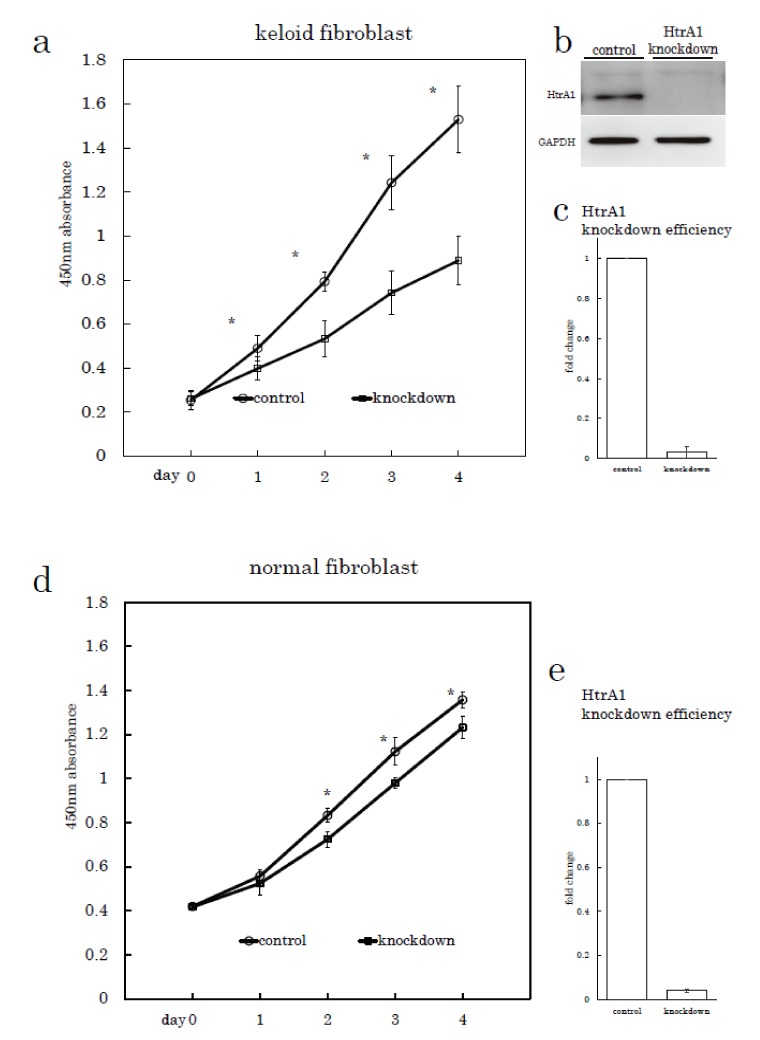
Proliferation rates of keloid fibroblasts and normal fibroblasts transfected with HtrA1 siRNA or control siRNA. Proliferation curves of keloid fibroblasts obtained from keloid sample No. 26 as shown in Table 1 (**a**), (*n* = 3) and normal fibroblasts from sample No. 8 (**d**) transfected with HtrA1 siRNA (knockdown) or control siRNA (control). The efficiency of HtrA1 knockdown in keloid fibroblasts was determined using western blot analysis (**b**) and quantitative PCR (**c**), (*n* = 3). The efficiency of HtrA1 knockdown in normal fibroblasts was similarly determined using quantitative PCR (**e**), *n* = 3. Cell proliferation was analysed using a colorimetric assay with a water-soluble tetrazolium salt as the substrate. Error bars represent standard deviations (*n* = 3). * *p* < 0.001.

To investigate role of HtrA1 in keloid pathogenesis, we examined whether HtrA1 affects cell proliferation by silencing *HtrA1* gene expression using specific small interfering RNA (siRNA). Keloid fibroblasts treated with *HtrA1* siRNA exhibited a proliferation rate significantly slower relative to those treated with control siRNA (Figure 6, Supplementary Figure S3). This effect with silencing HtrA1 was also observed in normal fibroblasts, but the inhibition effect was not as pronounced.

### 2.4. Additional HtrA1 in Culture Medium Stimulates Keloid Cell Proliferation

**Figure 7:**
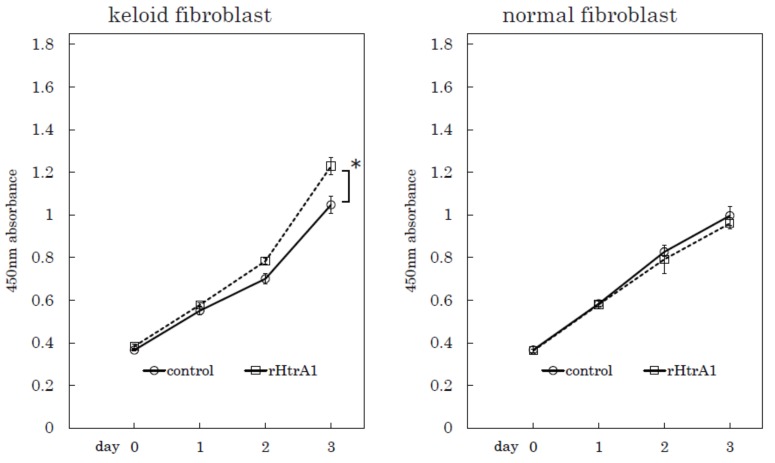
Proliferation rates of keloid fibroblasts and normal fibroblasts incubated with or without recombinant HtrA1. Proliferation curves of keloid fibroblasts obtained from sample No. 29 and normal fibroblasts from sample No. 8 as shown in Table 1, incubated with (rHtrA1) or without (control) recombinant HtrA1. *n* = 3, * *p* < 0.01.

To confirm the effect of HtrA1 on cell proliferation, we performed a proliferation assay on keloid fibroblasts with the addition of recombinant human HtrA1 in culture medium (Figure 7, Supplementary Figure S4). The addition of HtrA1 stimulated the proliferation of keloid fibroblasts, but not normal fibroblasts. These results suggest that HtrA1 plays an important role in keloid cell proliferation.

## 3. Discussion

In the present study, the expression of HtrA1 was strongly up-regulated in active keloid legions as analysed by in situ hybridization and immunohistochemical staining. Previous studies suggested that HtrA1 stimulates arthritis by digesting the ECM [14]. In arthritis, synovial fibroblasts produce abundant HtrA1, and HtrA1 digests cartilage ECM, including fibronectin, collagens, and proteoglycans. ECM fragments produced by HtrA1 digestion reportedly activate synovial fibroblasts and induce the remodelling of cartilage ECM. We propose that HtrA1 functions as a matrix protease that stimulates keloid development because the keloid matrix consists mainly of collagens, fibronectin, and proteoglycans, which are substrates for HtrA1. HtrA1 may degrade keloid matrix and accelerate ECM remodelling in keloid lesions. Matrix protein fragments produced by HtrA1 may activate keloid cells, leading to further progression of the disease. Consistent with this notion, we found HtrA1-knockdown inhibited the proliferation of keloid fibroblasts, and that recombinant HtrA1 added to the culture medium stimulated the proliferation of keloid fibroblasts. Interestingly, the inhibition or stimulation of proliferation with silencing or additional HtrA1 was clearly demonstrated in keloid fibroblasts, but not in normal fibroblasts. These results suggest that HtrA1 is a key molecule of keloid pathogenesis. The more keloid fibroblasts proliferate, the more matrix produced by keloid fibroblasts accumulates in keloid lesions.

Recently, Beaufort et al. reported that HtrA1 facilitates the transforming growth factor-beta (TGF-β) signalling through the processing of latent TGF-β binding protein (LTBP) [13]. Reduced TGF-β activity was observed in embryonic fibroblasts from HtrA1 knockout mice and skin fibroblasts from CARASIL patients caused by *HtrA1* mutations. These observations suggest a role of HtrA1 in facilitating TGF-β signalling. LTBP functions as a part of the large latency complex (LLC) that anchors TGF-β to the ECM [13,19,20,21,22]. Proteolysis of LTBP-1 results in its detachment from ECM, leading to TGF-β release and activation [20,23,24,25]. HtrA1 cleaves LTBP-1 in the fibronectin binding domain, and this processing occurs in a site-specific manner, distinct from other proteases previously reported [20,23,24,25]. TGF-β1 is overexpressed and activated in keloid lesions and plays a key role in keloid pathogenesis [26,27]. TGF-β1 stimulates production of abundant ECM including fibronectin and collagens. HtrA1 may facilitate keloid pathogenesis through the activation of TGF-β1 mediated by LTBP-1 cleavage.

HtrA1 has been reported to be a crucial molecule in AMD, a leading cause of irreversible blindness in the elderly [15,16]. AMD is accompanied with choroidal neovascularization and polypoidal choroidal vasculopathy. Analysis of HtrA1 transgenic mice indicated that increased HtrA1 is sufficient to cause hyper-vascularisation and degeneration of elastic laminae in choroidal vessels [15]. Zhang et al. demonstrated that HtrA1 promotes angiogenesis by regulating GDF6, a TGF-β family-protein, using HtrA1 knock-out mice [12]. As in AMD, abundant microvessels are observed in keloid lesions [9]. Thus, HtrA1 may play a role in keloid hypervascularity by modulating TGF-β family signalling.

Taken together, these observations suggest that HtrA1 contributes to the development of keloid lesions as matrix protease by remodelling keloid-specific ECM or cell surface molecules. HtrA1 may be useful as a target of keloid treatment, although further study is required.

## 4. Materials and Methods

### 4.1. Tissue Specimens

Between September 2007 and September 2013, 30 keloid patients (aged 16–75 years) and 12 unrelated patients (aged 31–88 years) undergoing surgical treatments were enrolled in this study. With approval from the Institutional Reviewing Board in the Kyoto University Faculty of Medicine (G61, the 14 December 2006), which adheres to the ethical standards as formulated in the Helsinki Declaration, written informed consent was obtained from all the patients. Keloid diagnosis was based on the clinical findings and definitive diagnosis was based on histopathologic data from the operative specimens [3,4]. The skin tissue samples were obtained as the surplus skin at the plastic surgery. Sample information is shown in Table 1.

### 4.2. Antibodies

Monoclonal anti-human HtrA1 antibody (MAB2916, R&D Systems, Minneapolis, MN, USA) was used for western blotting. The antibody used in immunohistochemical staining was developed in rabbits using a synthetic peptide corresponding to the C-terminal region of human HtrA1 as the immunogen.

### 4.3. In Situ Hybridization

For in situ hybridization, keloid and surrounding unaffected skin tissue specimens were obtained from the keloid patients at the time of surgical treatment. The specimens were fixed in 4% paraformaldehyde at 4 °C, embedded in paraffin, and Sections 6 μm in thickness were prepared. Deparaffinised sections were fixed in 4% paraformaldehyde in phosphate-buffered saline (PBS) for 15 min and washed with PBS. Sections were treated with 3 μg/mL proteinase K in PBS for 30 min at 37 °C, washed with PBS, refixed with 4% paraformaldehyde in PBS, washed again with PBS, and placed in 0.2 N HCl for 10 min. After washing with PBS, sections were acetylated by incubation in 0.1 M tri-ethanolamine-HCl (pH 8.0)/0.25% acetic anhydride for 10 min. After washing with PBS, sections were dehydrated through a series of ethanol solutions. Hybridization was performed with 1558-2066 of human *HtrA1* gene (Accession # NM_002775) at concentrations of 300 ng/mL in Probe Diluent-1 (Genostaff, Tokyo, Japan) at 60 °C for 16 h. After hybridization, sections were washed in 5× HybriWash (Genostaff) at 60 °C for 20 min, and in 50% formamide with 2× HybriWash at 60 °C for 20 min, followed by RNase treatment with 50 μg/mL RNase A in 10 mM Tris-HCl (pH 8.0)/1 M NaCl/1 mM EDTA for 30 min at 37 °C. Sections were then washed twice with 2× HybriWash at 60 °C for 20 min and twice with 0.2× HybriWash at 60 °C for 20 min. After treatment with 0.5% blocking reagent (Roche Diagnostics, Tokyo, Japan) in TBST (0.05 M Tris-HCl/0.15 M NaCl/0.05% Tween 20) for 30 min, sections were incubated for 2 h at room temperature with anti-DIG alkaline phosphatase conjugate (Roche Diagnostics) diluted 1:1000 with TBST. Sections were washed twice with TBST and then incubated in 100 mM NaCl/50 mM MgCl_2_/0.1% Tween20/100 mM Tris-HCl (pH 9.5). Colouring reactions were performed with NBT/BCIP solution (Sigma-Aldrich, Saint Louis, MO, USA) overnight, followed by washing with PBS. Sections were counterstained with Kernechtrot stain solution (Muto Pure Chemicals, Tokyo, Japan), dehydrated, and mounted with Malinol (Muto Pure Chemicals).

### 4.4. Immunohistochemical Analysis

All keloid and normal skin tissue specimens were obtained from the surgical treatment and fixed in 4% paraformaldehyde at 4 °C, and paraffin sections (3 μm) were prepared. Deparaffinised sections were incubated at 90 °C for 10 min in target retrieval solution (pH 9, 1:10, Dako, Glostrup, Denmark). After blocking endogenous peroxidase and non-specific protein binding activities, the sections were incubated with antibody against human HtrA1 (1:400) using LSAB^TM^2kit/HRP (Dako). After incubation with a peroxidase-conjugated anti-rabbit IgG antibody, sections were stained using a LSAB/HRP kit (Dako) and counterstained with haematoxylin. Microscopic images of sections were obtained by a Biorevo BZ-9000 microscope (Keyence, Osaka, Japan) and counting of total and stained fibroblasts was performed using ten microscopic fields at high-power (×400). The number of cells in the ten fields was determined. Stained fibroblasts per total fibroblasts were assumed as the proportion of HtrA1-positive cells.

### 4.5. Statistical Analysis

Significance of difference was analysed by the Student’s *t*-test. A *p*-value < 0.05 was taken as an indication of statistical significance.

### 4.6. Western Blot Analysis

Tissue samples were homogenized in RIPA buffer (Takara Bio, Otsu, Japan) containing protease inhibitors at 4 °C using a Polytron homogenizer (Kinematica, Luzern, Switzerland). Following centrifugation (12,000 rpm, 4 °C, 20 min), soluble proteins in the supernatant were separated by SDS-PAGE (gradient gels) and then blotted onto PVDF membranes. The membranes were blocked with 5% Block Ace (DS Pharma Biomedical, Osaka, Japan) in PBS containing 0.05% Tween 20 prior to incubation with anti HtrA1 antibody (1:500, R&D Systems). Specific antibody binding was detected by LAS-3000 (Fuji Photo Film, Tokyo, Japan).

### 4.7. Knockdown of HtrA1 Gene Expression and Cell Proliferation Assay

Keloid fibroblasts and normal fibroblasts were extracted by the explant method from surgical specimens. Briefly, tissues were cut into 1~2 mm^3^ pieces, placed into plastic tissue culture dishes, and cultured in Dulbecco’s modified Eagle’s medium (DMEM; Sigma-Aldrich, St. Louis, MO, USA) supplemented with 10% fetal calf serum, 10,000 U/mL penicillin G, and 10 mg/mL streptomycin sulphate. Cells were propagated at 37 °C, and semiconfluent cultures of fibroblasts were passaged by trypsinization up to twice prior to analysis. One day before transfection, keloid and normal fibroblasts were plated at 40% confluence at the 3rd passage in DMEM without antibiotics on 10-cm dishes, followed by transfection with *HtrA1* siRNA using Lipofectamine RNAiMAX Reagent, (Life Technologies, Carlsbad, CA, USA). After 48 h, the cell proliferation assay was performed using WST assay reagent (Nacalai Tesque, Kyoto, Japan). The expression levels of target gene and protein were analysed by real-time polymerase chain reaction (PCR) and western blot analysis, respectively. A proliferation assay of keloid and normal fibroblasts was also performed with or without the addition of recombinant human HtrA1 (R&D Systems, Minneapolis, MN, USA) to the culture medium.

### 4.8. Real-Time PCR Analysis

Total RNA was extracted from cells after the transfection using RNeasy Mini Kit (Qiagen, Venlo, The Netherlands). First-strand cDNA was synthesised using Prime Script RT Reagent Kit with gDNA Eraser (Takara Bio). RT-PCR was performed with cDNA using TaqMan Probe Assay (Applied Biosystems, Foster City, CA, USA). Glyceraldehyde-3-phosphate dehydrogenase was used as a housekeeping control gene. Relative expression was calculated by calibration curve method.

## 5. Conclusions

In summary, the expression of HtrA1 was revealed, especially in keloid active lesions, and the silencing of HtrA1 suppressed the proliferation of keloid fibroblasts. This effect of silencing HtrA1 was also observed in normal fibroblasts, but the inhibition effect was not so as pronounced. Moreover, the addition of recombinant HtrA1 in culture medium stimulated the proliferation of keloid fibroblasts but not normal fibroblasts. These results suggest that HtrA1 plays an important role in keloid cell proliferation and is a key molecule in keloid pathogenesis.

## Figures and Tables

**Table 1:** Samples used in this study.

Tissue Source	No.	Age	Sex	Region	HtrA1-Positve Cells (%) ^1^	Assays ^2^
Keloid	1	75	M	shoulder	18.83	A
	2	51	F	chest	12.40	A
	3	49	M	neck	38.74	A, B
	4	67	F	abdomen	43.52	A
	5	32	M	chest	24.77	A
	6	34	M	abdomen	40.64	A
	7	67	M	abdomen	32.80	A
	8	16	F	chest	41.90	A, C
	9	64	M	shoulder	25.82	A
	10	28	M	chest	24.85	A
	11	24	F	chest	28.36	A, C
	12	20	F	shoulder	35.43	A
	13	62	F	chest	12.68	A
	14	30	M	shoulder	35.34	A
	15	20	F	chest	48.37	A
	16	65	M	back	35.55	A
	17	38	F	chest	39.68	A
	18	39	F	abdomen		B
	19	75	M	chest		B
	20	21	M	back		B
	21	20	M	back		B
	22	31	M	shoulder		D
	23	20	F	shoulder		D
	24	20	F	chest		C, D
	25	58	F	shoulder		D
	26	24	M	chest		C
	27	41	F	abdomen		B
	28	27	M	chest		C
	29	17	F	chest		C
	30	24	F	chest		C
Normal skin	1	51	F	back	3.76	A, D
	2	45	F	abdomen	2.83	A
	3	47	F	shoulder	2.60	A
	4	51	F	thigh	2.13	A
	5	88	M	back		D
	6	49	F	abdomen		D
	7	51	F	abdomen		D
	8	52	F	chest		C
	9	53	F	chest		C
	10	40	F	abdomen		C
	11	31	F	abdomen		C
	12	52	F	chest		C

^1^ The percentage of HtrA1-positive cells was determined using immunohistochemical staining. ^2^ A, immunohistochemical staining; B, in situ hybridization; C, cell proliferation assay with silencing HtrA1 gene expression or with additional rHtrA1; D, western blotting.

## Supplementary Materials

Supplementary materials can be found at http://www.mdpi.com/1422-0067/19/5/1275/s1.

## Abbreviations

HtrA1	High Temperature Requirement Factor A1
