# Supplementary material for: HtrA1 Is Specifically Up-Regulated in Active Keloid Lesions and Stimulates Keloid Development
Source: Int J Mol Sci. 2018 Apr 24;19(5):1275. doi: 10.3390/ijms19051275 (PMC5983720; doi:10.3390/ijms19051275)
Supplement: Supplementary file 1 [file ijms-19-01275-s001.docx]

HtrA1 is Specifically Up-Regulated in Active Keloid Lesions and Stimulates Keloid Development

**Supplemental Figure S1.** In situ hybridization of *HtrA1* mRNA in keloid lesions. Sections were hybridized with HtrA1 antisense probe (**a**, **c**, and **e**) or sense probe (**b**, **d**, and **f**). Sections of keloid lesions from three different patients were analysed, and sections shown in **a** and **b** were obtained from the same patient (patient no. keloid-19 in Table 1). Sections in **c** and **d** (patient no. keloid-3 in Table 1), or **e** and **f** (patient no. keloid-20 in Table 1) were obtained from two other patients. Positive signals are visualized in blue. Scale bar = 50 µm

**Supplemental** **Figure S2.** Immunohistochemical staining of HtrA1 protein in keloid lesions. Sections were stained with anti HtrA1 antibody (**a**, **c**, and **e**) or without the primary antibody (**b**, **d**, and **f**). Sections of keloid lesions from three different patients were analysed, and sections shown in **a** and **b** were obtained from the same patient (patient no. keloid-3 in Table 1). Sections in **c** and **d** (patient no. keloid-4 in Table 1), or **e** and **f** (patient no. keloid-5 in Table 1) were obtained from two other patients. Positive signals are visualized in brown. Scale bar = 50 µm


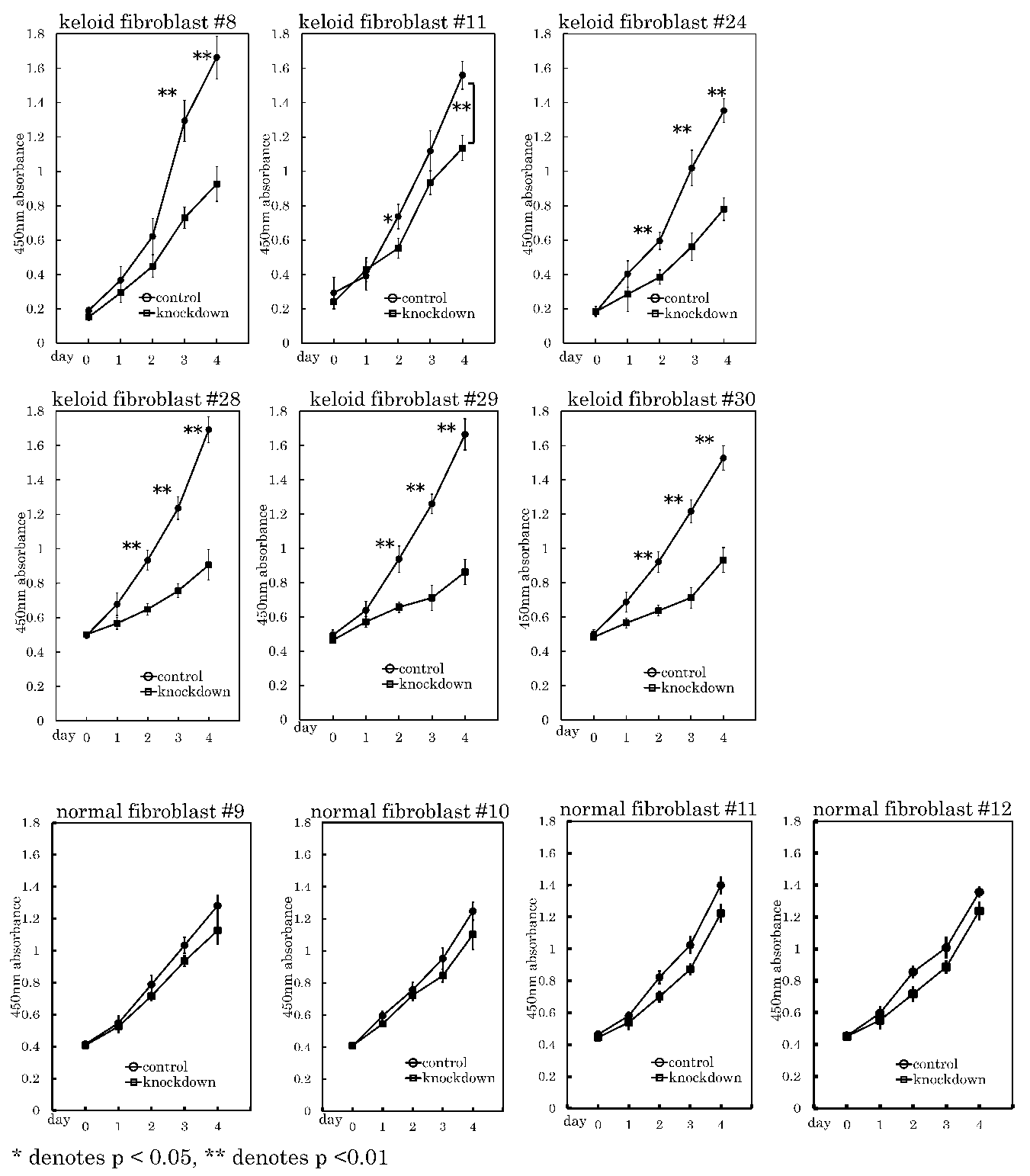


**Supplemental** **Figure S3.** Proliferation rates of keloid fibroblasts and normal fibroblasts transfected with HtrA1 siRNA (knockdown) or control siRNA (control). Proliferation curves of keloid fibroblasts obtained from sample Nos. 8, 11, 24, 28, 29 and 30 as shown in Table 1 and normal fibroblasts from sample Nos. 9 to 12 transfected with HtrA1 siRNA (knockdown) or control siRNA (control). *p* < 0.001.

**Supplemental** **Figure S4.** Proliferation rates of keloid fibroblasts and normal fibroblasts incubated with or without recombinant HtrA1. Proliferation curves of keloid fibroblasts obtained from sample Nos. 28 and 30 as shown in Table 1 and normal fibroblasts from sample Nos. 9 and 10 incubated with (rHtrA1) or without (control) recombinant HtrA1. *, *p* < 0.05; **, *p* < 0.001.
